# Integrated microbiome and metabolome analysis reveals novel urinary microenvironmental signatures in interstitial cystitis/bladder pain syndrome patients

**DOI:** 10.1186/s12967-023-04115-5

**Published:** 2023-04-19

**Authors:** Zhenming Zheng, Jintao Hu, Wenshuang Li, Kaiqun Ma, Caixia Zhang, Kuiqing Li, Yousheng Yao

**Affiliations:** 1 Department of Urology, Sun Yat-Sen Memorial Hospital, Sun Yat-Sen University, Guangzhou, China; 2 Department of Urology, The First Affiliated Hospital of Anhui Medical University, Hefei, China; 3 Department of Urology, Shantou Central Hospital, Shantou, China

**Keywords:** Interstitial cystitis, Bladder pain syndrome, Microbiome, Metabolomics, Urine

## Abstract

**Background:**

The pathogenesis of interstitial cystitis/bladder pain syndrome (IC/BPS) has not been elucidated, but urinary microorganisms and metabolites have been shown to be closely associated with the inflammatory response of IC/BPS. Nevertheless, the exact mechanisms related to this response have not been clarified.

**Methods:**

16S rRNA sequencing and untargeted metabolomics techniques were used to analyse the urinary microbial and metabolite profiles of 30 IC/BPS patients and 30 healthy controls, and correlation analyses were performed to explore the mechanisms by which they might be involved in the inflammatory response of IC/BPS.

**Results:**

Twenty-eight differential genera, such as *Lactobacillus* and *Sphingomonas,* were identified. A total of 44 differential metabolites such as 1,3,7-trimethyluric acid and theophylline were screened. The abundance of *Lactobacillus* and *Escherichia-Shigella* was significantly higher in the urine of female IC/BPS patients and healthy controls compared to males, while *Bacteroides* and *Acinetobacter* were lower than in males. The results of the Pearson correlation analysis suggested that differential microorganisms may influence the composition of metabolites. The *Lactobacillus* genus may be a protective bacterium against IC/BPS, whereas *Sphingomonas* may be a pathogenic factor. The differential metabolite theophylline, as an anti-inflammatory substance, may downregulate the inflammatory response of IC/BPS.

**Conclusions:**

This study revealed microbial and metabolite profiles in the urine of IC/BPS patients versus healthy controls in both males and females. We also found some microorganisms and metabolites closely related to the inflammatory response of IC/BPS, which provided directions for future aetiological and therapeutic research.

**Supplementary Information:**

The online version contains supplementary material available at 10.1186/s12967-023-04115-5.

## Introduction

Interstitial cystitis/bladder pain syndrome (IC/BPS) is a clinical syndrome characterized by symptoms such as urinary frequency, urgency, and bladder and/or pelvic pain [1]. IC/BPS patients often experience discomforts such as sleep dysfunction, depression, anxiety, and sexual dysfunction, which seriously affect the patient’s daily life and work and are associated with a heavy societal burden [2]. The pathogenesis of IC/BPS is unclear, and the curative effects of existing treatment regimens are unsatisfactory. Therefore, it is particularly important to clarify the aetiology of IC/BPS and explore new treatment methods.

With the advent of new technologies such as 16S rRNA high-throughput sequencing, metagenomic sequencing, and enhanced quantitative urine culture (EQUC), a unique urinary microbiota (UM) undetectable by routine urine testing and common urine bacterial culture has been discovered, providing the possibility to study the association between urinary tract microbiota and diseases [3–5]. Previous studies have found that there are significant differences in the urinary microbiota between healthy people and patients with lower urinary tract dysfunction and urinary tract tumours [6–9]. Previous studies on the urinary microbiota of IC/BPS patients have been inconsistent [10]: some studies have found that the diversity of their urinary microbiota, especially *Lactobacillus*, is low and that women with a lower abundance of *Lactobacillus* in their urine have an increased risk of IC/BPS-related symptoms [11], while other studies have reported no significant difference in the abundance and diversity of urinary flora in IC/BPS patients and healthy people [12]. In most previous studies, the characteristics of the urinary microflora in only female IC/BPS patients were assessed, and few studies on the urinary microbes in male IC/BPS patients have been conducted.

The metabolome refers to the collection of all small-molecule metabolites in a cell, tissue, or organ during a specific physiological period. Urine is the body fluid closest to the urinary tract and is an important specimen reflecting renal function and urinary system disease status. Therefore, it is of great significance to study the correlation between urinary metabolites and urinary system diseases. The study by Kim J et al. found that the expression levels of phenylalanine, purine, 5-oxo proline, and 5-hydroxyindoleacetic acid were significantly different in IC/BPS patients [13]. Fukui, Y. et al. [14] found that the content of phenylacetylglutamine (PAGN) in the urinary metabolites of IC/BPS patients was significantly higher than that of the control group and identified it as a urinary marker for the diagnosis of IC/BPS. The results from previous studies on IC/BPS metabolomics have been varied, as urinary metabolites are susceptible to influences including metabolites from food, drugs, nutrients, and bacteria. Therefore, the results obtained by only analysing the correlation of urinary metabolites with the disease will have large individual differences. Microorganisms in urine are also directly involved in the complex metabolic processes of the human body. Consequently, combining urinary microbes and urinary metabolites for analysis may lead to more clinically meaningful results.

The current study focused on the urinary microenvironment of IC/BPS patients. 16S rRNA high-throughput sequencing and untargeted metabolomics technology were used to explore the characteristics of urinary microbiota and metabolites in patients with IC/BPS, and the correlation analysis of differential microorganisms and metabolites was performed to analyse their effects on the inflammatory response of IC/BPS patients. This study provides new insights into the aetiology and treatment of IC/BPS.

## Materials and methods

### Study subjects and inclusion, and exclusion criteria

A total of 30 patients who were diagnosed with IC/BPS by a professor of urology in the Department of Urology of Sun Yat-sen Memorial Hospital of Sun Yat-sen University from June 2020 to June 2021 and 30 healthy volunteers (control group) matched for age and sex during the same period were selected as research subjects. All participants completed the following assessments: interstitial cystitis symptom index (ICSI), interstitial cystitis problem index (ICPI), visual analogue scale for pain (VAS), patient symptom questionnaire for pelvic pain, urgency and frequency (PUF), self-rating of anxiety scale (SAS), and self-rating depression scale (SDS). Additionally, the demographic characteristics of the two groups were collected. The diagnostic criteria for IC/BPS patients included in this study were based on the diagnostic criteria established by the National Institute of Diabetes and Digestive and Kidney Diseases (NIDDK) [15]. Both the IC/BPS group and the healthy control group were excluded from urinary tract infection by routine urine test or urine bacterial culture, and those with a history of urinary tract infection or antibiotic use within the past 3 months were also excluded. Long-term consumption of probiotics or other drugs, and those with metabolic diseases such as hyperthyroidism and diabetes could not participate in this study. All participants voluntarily participated and signed informed consent, and this study was approved by the ethics committee of Sun Yat-sen Memorial Hospital of Sun Yat-sen University.

### High-throughput 16S ribosomal RNA gene sequencing

The urinary meatus and perineum of all participants were disinfected with 0.5% iodophor, and 20 ml of clean midstream urine was collected. Samples were centrifuged immediately after collection (10 min, 4 °C, 1600 g), the separated supernatant and urine sediment were stored separately, urine sediment was used for 16S rRNA sequencing and supernatant for subsequent metabolomic analysis, both stored at −80 °C until use. Total DNA was extracted using the HiPure stool DNA kit (New England Biolabs) according to the manufacturer's protocol, and a NanoDrop (NanoDrop ND-2000, United States) was used to check the concentration and purity of DNA. The V3-V4 region was used as the amplification region for PCR amplification of the 16S rRNA gene. The primer sequences were 341F (5'-CCTACGGGNGGCWGCAG-3') and 806R (5'-GGACTACHVGGGTATCTAAT-3'), with a length of approximately 466 bp. The AxyPrep DNA Gel Extraction Kit (Axygen Biosciences, Union City, CA, USA) was used to purify the amplification products and quantify them using the ABI StepOnePlus Real-Time PCR System (Life Technologies). The PCR products from different samples were homogenously mixed to construct an Illumina library. Paired-end sequencing (2 × 250) of the amplicon library was performed on the Illumina HiSeq 2500 platform (Illumina, San Diego, USA) according to standard protocols.

After sequencing was completed, the data were processed for splicing, chimaera removal, and quality control to obtain the operational taxonomic unit (OTU) sequences and OTU abundance information. Subsequently, taxonomic assignment and PICRUSt (version 2.1.4) functional prediction of 16S rRNA reads was performed with reference to the SILVA (Version 132) database. The differences in microbiota abundance between the two groups were compared by Welch's t test and the Wilcoxon rank sum test. Spearman correlation analysis was used to analyse the correlation between clinical characteristics and microbiota.

### Liquid chromatography coupled with mass spectrometry

Non-targeted metabolomics analyses were performed with reference to those described in previous studies [16, 17]. In brief, to extract the metabolites, an extraction solution (volume ratio of methanol: acetonitrile: water = 2:2:1) was added to the urine samples, and the sample vortexed for 30 s, sonicated in an ice-water bath for 10 min, incubated for 1 h at −40 °C, and centrifuged (15 min, 12000 rpm, 4 °C). Mobile phase A and Mobile phase B consisted of H_2_O and acetonitrile, both containing 0.1% of formic acid. The temperature of the autosampler was set at 4 °C, the column temperature at 35 °C, the injection volume at 2 μl, and the mobile phase flow rate at 0.5 mL/min. Chromatographic separation of metabolites was performed on a Waters ACQUITY UPLC BEH Amide column using a Vanquish (Thermo Fisher Scientific) ultra-performance liquid chromatograph. Primary and secondary mass spectrometry (MS_1 and MS_2) data were acquired via a Thermo Q Exactive HFX mass spectrometer and control software (Xcalibur, Thermo, version: 4.0.27, Thermo). The raw data were converted to mzXML format using ProteoWizard and then analysed. Multivariate statistical analysis (OPLS-DA, VIP ≥ 1, T test P < 0.05) was used to identify differential metabolites in the urine of the two groups. According to the metabolite qualitative results, the metabolites corresponding to compound IDs were found from the KEGG database, and metabolite function annotation and pathway enrichment analysis were performed on the differential metabolites through KEGG. The metabolic pathways with a Q ≤ 0.05 were considered to have significant differences.

### Correlation analysis of differential microorganisms and differential metabolites in urine

The differential genera and differential metabolites identified in 16S sequencing and untargeted metabolomic analysis were extracted, and Pearson correlation analysis was used to explore the correlation between differential flora and differential metabolites. The Pearson correlation coefficients for the differential genera and differential metabolites in urine were calculated using the R cor. test function. r took values in the range [−1, 1], and p values were calculated based on the Fisher-Z transformation. *P* < 0.05 was considered a statistically significant difference, and correlation heatmaps and network plots were drawn.

### Statistical analysis

Statistical analysis was performed using R 4.1.3 and the statistical package for the social sciences (SPSS, Version 25, USA). Normally distributed numerical variables were described by the mean ± standard deviation (X ± s), and a two-tailed Student's t test was used to compare the differences between groups. Nonnormally distributed data were described by quartiles, and the Wilcoxon test was used to analyse the intergroup differences. Categorical variables were described as percentages, and the chi-square test or Fisher’s test was used for the comparison. The correlation analysis between differential metabolites and differential microorganisms was performed by Pearson correlation analysis, and a P < 0.05 was considered to be statistically significant.

## Results

### Demographic and clinical characteristics of participants

A total of 30 IC/BPS patients and 30 age- and sex-matched healthy controls were included in this study. There were no significant differences in basic clinical information such as age, sex, marital status, fertility status, and smoking or not between the two groups of participants (*P* > 0.05). The BMI of the IC/BPS group was lower than that of the healthy control group (20.83 vs. 22.87, *P* < 0.01). IC/BPS-related clinical symptom scores were determined for all participants. In the IC/BPS group, the VAS (2.40 vs. 0.30), PUF (16.70 vs. 4.33), ICSI (13.80 vs. 1.87), ICPI (11.37 vs. 1.57), SDS (53.23 vs. 40.27), and SAS (54.20 vs. 41.78) scores were significantly higher than those in the control group (*P* < 0.01), as shown in Table 1.

**Table 1:** Demographic characteristics and symptom scores of IC/BPS and control groups

Characteristics	IC/BPS group (n = 30)	Control group (n = 30)	P-value
Sex			0.79
Male	11	10	
Female	19	20	
Age	40.07 ± 12.96	39.13 ± 13.24	0.78
BMI	20.83 ± 2.44	22.87 ± 3.05	< 0.01
Marital status			0.35
Unmarried	5	8	
Married	25	22	
Fertility status			0.77
No children	7	8	
One or more children	23	22	
Smoking			0.32
Presence	7	4	
Absence	23	26	
VAS	2.40 ± 0.86	0.30 ± 0.47	< 0.01
PUF	16.70 ± 4.10	4.33 ± 1.49	< 0.01
ICSI	13.80 ± 2.01	1.87 ± 0.86	< 0.01
ICPI	11.37 ± 1.63	1.57 ± 0.97	< 0.01
SDS	53.23 ± 3.79	40.27 ± 3.87	< 0.01
SAS	54.20 ± 4.60	41.78 ± 4.20	< 0.01

BMI: Body Mass Index, VAS: Visual Analogue Scale for Pain, PUF: Patient Symptom Questionnaire for Pelvic Pain, Urgency and Frequency, ICSI: Interstitial Cystitis Symptom Index, ICPI: Interstitial Cystitis Problem Index, SDS: Self-Rating Depression Scale, a standard score greater than 53 is considered depression, 53-62 is mild depression, 63-72 is moderate depression, and more than 72 is severe depression, SAS: Self-Rating Anxiety Scale, a standard score greater than 50 is considered anxiety, 50-60 is mild anxiety, 61-70 is moderate anxiety, and 70 or more is severe anxiety

#### Alterations in bacterial diversity in IC/BPS patients

**Fig. 1:**
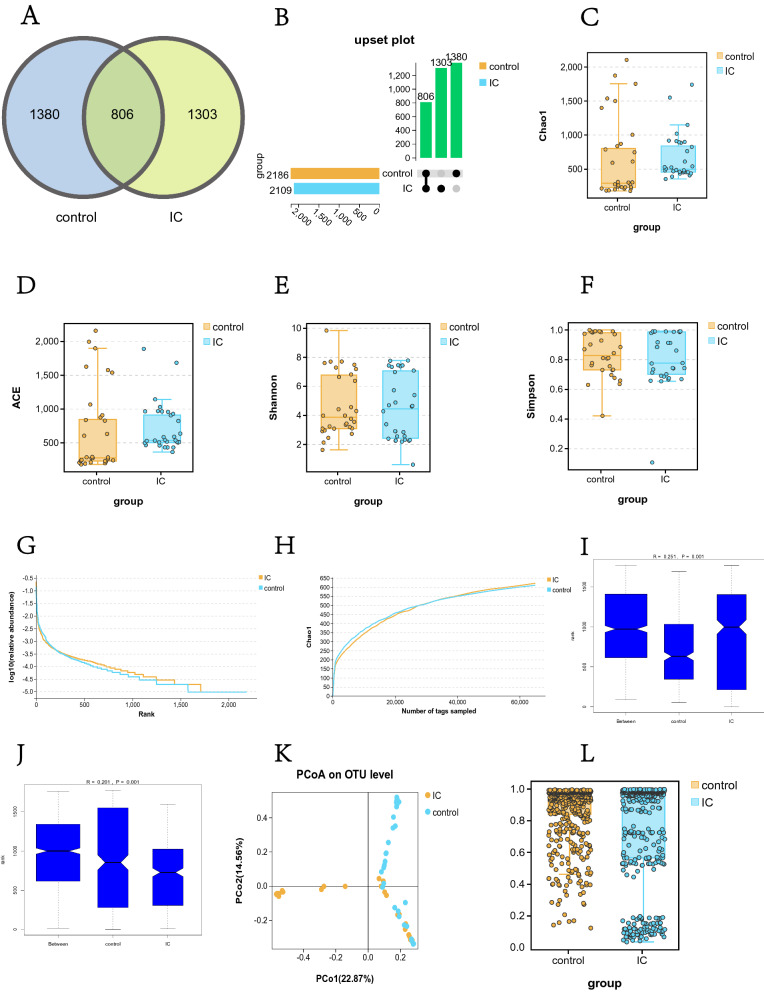
Comparison of urinary microbial diversity between the IC/BPS group and the control group. **A** Venn diagram of OTU clustering and **B** UpSet plot showing 1380 OTUs in the control group, 1303 OTUs in the IC/BPS group, and 806 OTUs shared by the two groups (**C**: Chao1 index, **D**: ACE index, **E**: Shannon index, **F**: Simpson index). -diversity analysis indicated that there was no significant difference in -diversity between the two groups (P > 0.05). **G** The close proximity of the two rank abundance curves further showed that there was no significant difference inβ-diversity between the two groups. **H** The dilution curve tended to be flat, indicating that the sequencing depth was sufficient. **I** Weighted ANOSIM test and **J** unweighted ANOSIM test, R > 0 and P = 0.001 indicated that the intergroup difference was greater than the intragroup difference, which means that our grouping was valid. **K** PCoA plot showing the similarity of the urinary microbiota between the two groups. **L** The β-diversity statistical test results indicated that the β-diversity between the IC/BPS group and the control group was significantly different (Bray distance index, OTU level, Welch's t test, *P* < 0.01)

Detailed 16S sequencing information for IC/BPS and control patients are provided in Additional file 1: Table S1. The results of OTU cluster analysis showed that there were 1380 OTUs in the control group, 1303 OTUs in the IC/BPS group, and 806 OTUs shared by the two groups. There was no significant difference in the number of OTUs between the two groups (Fig. 1A, B). The results of alpha (α) diversity analysis (Fig. 1C, D, E, F) showed that there was no significant difference in the -diversity of urine between the IC/BPS group and the control group (Chao1 index 679.3 vs. 653.0, ACE index 723.9 vs. 682.5, Shannon index 4.5 vs. 4.7 and Simpson index 0.8 vs. 0.8), *P* > 0.05, indicating that there was no significant difference in the abundance and uniformity of urinary microflora between the two groups. The rank abundance curve was used to reflect the taxonomic richness and evenness contained in the samples. Figure 1G shows that the rank abundance curves of the two groups were adjacent to each other in both the horizontal and vertical directions, further indicating that there was no significant difference in urinary α-diversity between the two groups. Both dilution curves in Fig. 1H have flattened, indicating that our sequencing depth is reasonable and has covered the vast majority of species in the sample. The beta (β) diversity analysis results (Fig. 1I, J) showed R > 0, indicating that the intragroup difference was less than the intergroup difference; that is, the grouping was effective. The P value of the comparison between groups was 0.001, indicating that the microbiota structure of IC/BPS patients was significantly different from that of healthy people. The PCoA plot in Fig. 1K further clearly shows the difference in microbiota distribution between IC/BPS patients and healthy controls. The results of the β-diversity statistical test (Fig. 1L) further confirmed that there was a significant difference in β-diversity between the IC/BPS group and the control group (OTU level, Welch's t test, *P* < 0.01).

#### Alterations in the composition of urinary microflora in patients with IC/BPS

**Fig. 2:**
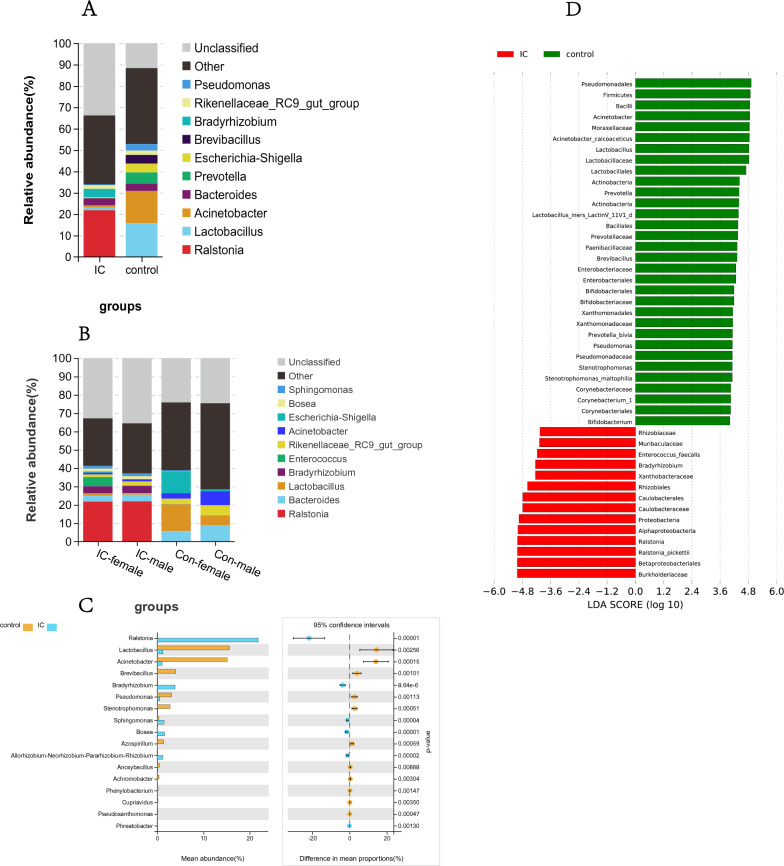
Urinary flora composition and differential microbial analysis of the IC/BPS group and control group. **A** Stacked map of differential genus distribution between the IC/BPS group and control group, **B** stacked map of differential genus distribution of males and females, **C** histogram of differential genus abundance between the IC/BPS group and control group (*P* < 0.01), **D** LEfSe analysis histogram (LDA score > 4.0, Wilcoxon rank-sum tests,* P* < 0.05)

We counted and analysed the microbial community composition of the IC/BPS group and control group at each bacterial taxonomic level (phylum, class, order, family, genus, species). Limited by the sequencing depth of 16S rRNA, many microorganisms at the species level could not be identified, so we focused on the analysis of microorganisms at the genus level. The species stacking diagram (Fig. 2A) visually shows the abundance and proportion of different bacterial genera in both the IC/BPS group and the control group. Additionally, the content of the urinary microbiota in the two groups from high to low at the genus level was *Ralstonia, Lactobacillus, Acinetobacter, Prevotella and Escherichia-Shigella, Brevibacillus, Bradyrhizobium, Rikenellaceae,* and *Pseudomonas.* There were significant differences in species composition and abundance between the two groups at the genus level. The abundance of *Ralstonia* in the IC/BPS group was significantly higher than that in the control group, while the abundance of *Lactobacillus* and *Acinetobacter* in the IC/BPS group was significantly lower than that in the control group.

The results of differential genus analysis between males and females (Fig. 2B) showed that at the genus level, there were significant differences in microbial abundance between males and females in both the IC/BPS group and the control group. The abundances of *Lactobacillus* and *Escherichia-Shigella* were significantly higher in females than in males in the IC/BPS group and control group, while the abundances of *Bacteroides* and *Acinetobacter* were higher in males than in females. We also found that there were significant differences in microbial composition and abundance between the IC/BPS group and the control group of the same sex. For example, the abundance of *Lactobacillus* in the control group was significantly higher than that in the IC/BPS group, while *Ralstonia* in the IC/BPS group was higher than that in the control group, which further indicates that the urinary flora of IC/BPS patients has been significantly changed.

Species whose sum of abundance in all samples was less than 0.1% were filtered out, and then the difference analysis (Welch's *t* test,* P* < 0.01) was performed between the IC/BPS group and the control group, and the results are shown in Fig. 2C. At the genus level, the abundances of *Lactobacillus, Acinetobacter, Brevibacillus, Pseudomonas, Stenotrophomonas*, and *Azospirillum* in the IC/BPS group were significantly lower than in the control group, while the abundances of *Ralstonia, Bradyrhizobium, Sphingomonas,* and *Bosea* were significantly higher than those of the control group (*P* < 0.01). LEfSe analysis results (Fig. 2D) further clearly showed the differences in microbiota between the two groups.

#### Potential functions of differential urinary microbes between the IC/BPS and control groups

**Fig. 3:**
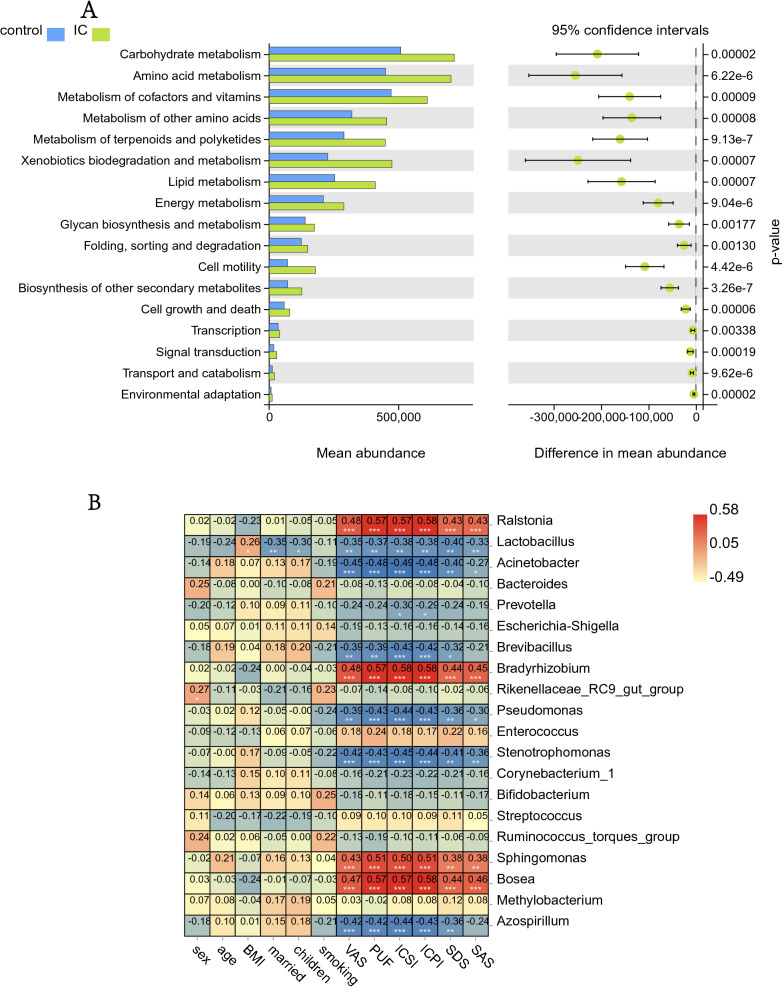
Differential microbial function analysis of the IC/BPS group and control group. **A** PICRUSt2 functional prediction analysis compared functional differences between the IC/BPS and control groups for differential microorganisms (Welch's t test, *p* < 0.05), with the Y-axis referring to a different function or pathway, the left half of the X-axis referring to the mean functional abundance, and the right half of the horizontal coordinates referring to the confidence interval range for the difference in functional abundance between groups. **B** Correlation analysis results of differential microorganisms with demographic characteristics and clinical symptoms (blue means negative correlation, red means positive correlation, *P < 0.05, **P < 0.01, ***P < 0.001)

According to the species annotation and abundance information of OTUs, we predicted the gene functions and pathways that might be affected by the differential microorganisms. The results (Fig. 3A) showed that IC/BPS group and control group had significant differences in the following functions and pathways due to changes in the urinary microbiome (*P* < 0.01): carbohydrate metabolism, amino acid metabolism, lipid metabolism, energy metabolism, cofactor, and vitamin metabolism, nucleic acid synthesis and decomposition, production of metabolites, cell growth and death, environmental adaptation, signal transduction, etc.

#### Association of clinical characteristics with urinary microbes

To study the correlation between age, sex, fertility status, BMI, and other demographic characteristics and clinical symptoms of IC/BPS patients and healthy controls with their urinary flora, we carried out a correlation analysis between differential bacteria and environmental factors (demographic characteristics and clinical symptoms). The correlation heatmap (Fig. 3B) shows the correlation of specific environmental factors with each differential strain. We found that ICSI, ICPI, PUF, and VAS scores correlated strongly with the abundance of differential genera. The abundances of *Ralstonia, Bradyrhizobium, Sphingomonas*, and *Bosea* were positively correlated with SDS, ICSI, ICPI, PUF, and VAS scores, while demographic characteristics such as age, sex, and fertility status had relatively small impact.

#### Profile and alterations of urinary metabolites in IC/BPS patients

**Fig. 4:**
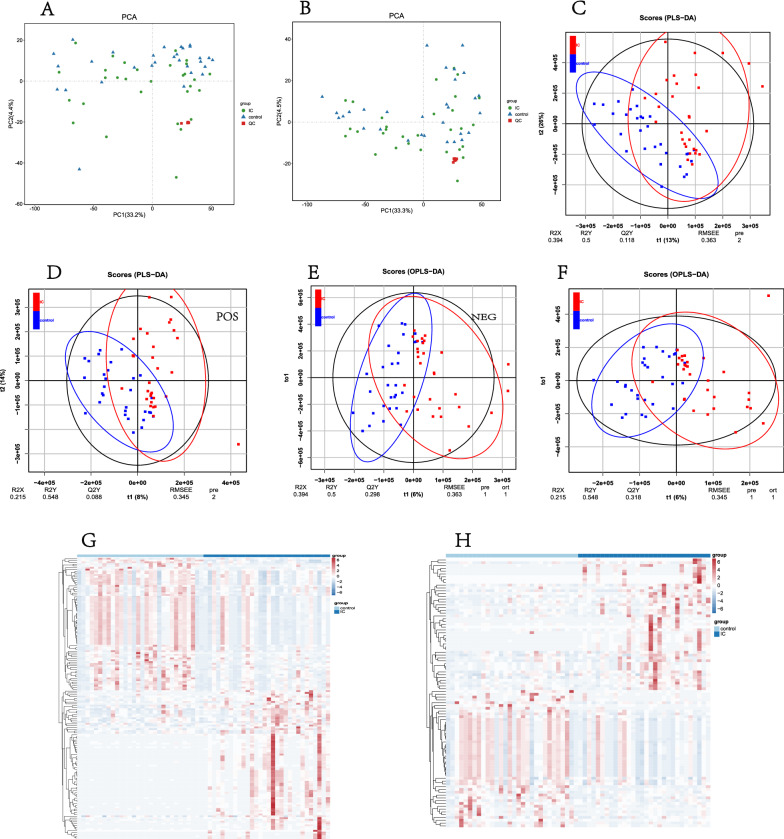
Identification of urinary metabolites in the IC/BPS and control groups. **A**, **B** In the quality control PCA graphs of the IC group and the control group, the red QC samples are densely distributed, indicating that the quality is stable and that these results are reliable. **C**, **D** PLS-DA score map of the IC/BPS group and control group based on the urinary metabolic profile; **E**, **F** OPLS-DA model score map of differential metabolites in the IC/BPS group and control group. **G**, **H** Heatmap of metabolite clustering in the IC/BPS group and control group (**A**, **C**, **E**, and **G** are positive ion models; **B**, **D**, **F**, and **H** are negative ion models)

**Fig. 5:**
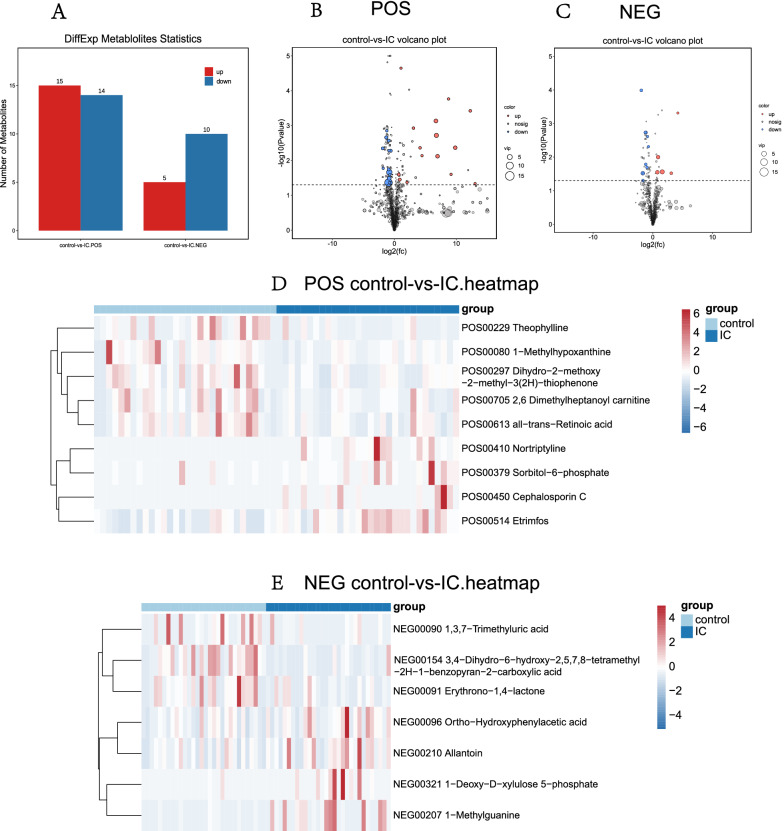
Comparison of differential urinary metabolites between the IC/BPS group and the control group. **A** A total of 44 differential metabolites were identified, with positive ions on the left and negative ions on the right. Red and blue represent the number of metabolites that were upregulated and downregulated in the IC/BPS group compared to the control group, respectively. **B**, **C** Volcano plots of differential urinary metabolites between the IC/BPS group and the control group, **D**, **E** heatmaps of differential urinary metabolites (**B**, **D**: positive ion model, **C**, **E**: negative ion model)

First, we performed a quality control analysis, and the red QC samples in the PCA plot were densely distributed, indicating that our research results were reliable (Fig. 4A, B). The metabolite cluster heatmap (Fig. 4G, H) showed the metabolite expression specific to each sample. Partial least-squares discriminant analysis (PLS-DA) and orthogonal partial least squares-discriminant analysis (OPLS-DA) results (Fig. 4C, D, E, F) clearly showed the distribution of urinary metabolites in the two groups of patients. Then, we constructed an OPLS-DA model (VIP ≥ 1, T test P < 0.05) to identify differential metabolites. Of the 2180 metabolites with known names identified in the samples (Additional file 3: Table S3 and Additional file 4: Table S4), 44 metabolites were significantly different in the urine of the IC/BPS and control groups (Additional file 1: Table S2), of which 24 were lower in the IC/BPS group than in the control group and 20 were higher in the IC/BPS group than in the control group (Fig. 5A). The enriched anionic differential metabolites were as follows: ortho-hydroxyphenyl acid, allantoin, 3,4-dihydro-6-hydroxy-2,5,7,8-tetramethyl-2H-1-benzopyran-2-carboxylic acid, 1-deoxy-d-xylulose 5-phosphate, erythrono-1,4-lactone, 1,3,7-trimethyluric acid, and 1-methylguanine. Positive ion differential metabolites were as follows: 1-methylhypoxanthine, 2,6 dimethylheptanoyl carnitine, all-trans-retinoic acid, cephalosporin C, dihydro-2-methoxy-2-methyl-3(2H)-thiophene, etrimfos, nortriptyline, sorbitol-6-phosphate, and theophylline. The heatmap and volcano map of the differential metabolites visually showed the expression of specific differential metabolites in the IC/BPS group and the control group (Fig. 5B, C, D, E).

#### Potential functions of differential metabolites

**Fig. 6:**
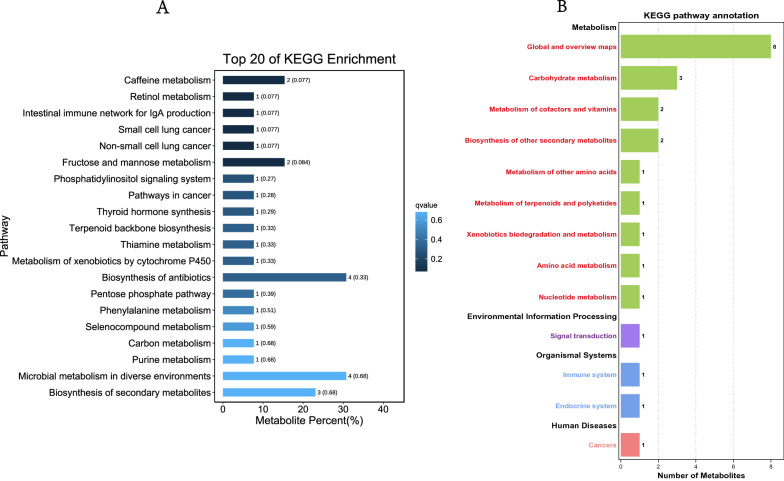
Functional annotation and pathway enrichment analysis of differential metabolites between the IC/BPS group and the control group. **A** Differential metabolite-related functions, **B** differential metabolite-related pathways

To explore the functions of differential metabolites and the metabolic pathways that they may participate in, Kyoto Encyclopedia of Genes and Genomes (KEGG) functional annotation and metabolic pathway enrichment analysis were conducted for differential metabolites. The results (Fig. 6) showed that these differential metabolites may be related to the synthesis and metabolism of amino acids, nucleic acids, purines, carbohydrates, the pentose phosphate pathway, thyroid hormone synthesis, signal transduction, and other metabolic pathways as well as endocrine and immune induction of tumour occurrence and progression.

### Correlation analysis of differential microorganisms and differential metabolites in urine

**Fig. 7:**
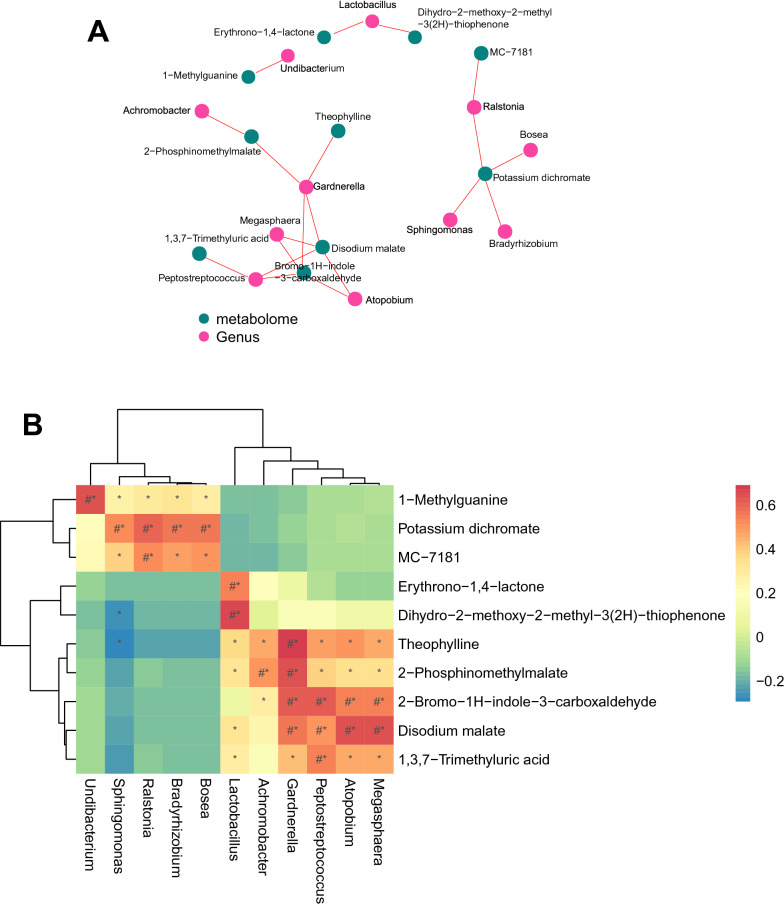
Correlation analysis of differential bacterial genera and differential metabolites. **A** Correlation network diagram, red circles represent correlated differential bacterial genera, and green circles represent correlated differential metabolites. **B** Correlation heatmap, the horizontal axis is the differential bacterial genera, and the vertical axis is the differential metabolites, red means positive correlation, blue means negative correlation (# represents the absolute value of correlation coefficient |r|≥ 0.5, and * represents *P* < 0.05)

Since it is difficult to accurately identify microorganisms at the species level with 16S sequencing, we focused on the correlation between differential microorganisms and differential metabolites at the genus level. At the genus level, a total of 11 differential microorganisms associated with differential metabolites were identified, of which the genera in the IC/BPS group were significantly higher than those in the control group: *Ralstonia, Bradyrhizobium, Sphingomonas, Bosea,* and *Undibacterium.* The genera that were significantly lower in the IC group than in the control group were as follows: *Lactobacillus, Gardnerella, Achromobacter, Atopobium, Megasphaera, and Peptostreptococcus.* A total of 10 differential metabolites were identified, and the metabolites in the IC/BPS group were significantly lower than those in the control group: dihydro-2-methoxy-2-methyl-3(2H)-thiophene, 2-phosphinomethylmalate, disodium malate, 2-bromo-1H-indole-3-carboxaldehyde, erythroprono-1,4-lactone, 1,3,7-trimethyluric acid, and theophylline. The metabolites in the IC/BPS group were significantly higher than those in the control group: potassium dichromate, MC-7181, and 1-methylguanine. We found that *Sphingomonas* was negatively correlated with the contents of the differential metabolites dihydro-2-methoxy-2-methyl-3(2H)-thiophene and theophylline, while the abundance of other differential genera and differential metabolites were positively correlated. We plotted correlation networks (Fig. 7A) to identify differential microbial-metabolite pairs with absolute values of correlation coefficients |r|> 0.5 and then plotted correlation heatmaps (Fig. 7B) for these differential microbes and metabolites. (# represents the absolute value of correlation coefficient |r|≥ 0.5, and * represents *P* < 0.05).

## Discussion

The commensal microorganisms and pathogenic microorganisms in the human urinary system together constitute the microecosystem of the human urinary system. Both an increase in pathogenic microorganisms and a decrease in probiotics may lead to bladder disease through a variety of mechanisms. Urinary microbiota may produce small molecules that interact with the nervous system and play a role in the regulation and maintenance of bladder function. Commensal bacteria may form a protective barrier in urothelial cells, produce antibacterial compounds, and degrade harmful products, thereby defending against the invasion of foreign urinary pathogens, and pathogenic microorganisms may directly lead to the occurrence of bladder inflammation or may attack commensal bacteria, resulting in an imbalance of the flora in the urinary system and indirectly leading to the occurrence of bladder diseases. Urinary metabolites not only reflect the metabolic state of the human body but also may convey the pathophysiological information of urinary system-related diseases. Because urinary metabolites are easily affected by food, drugs, and nutrients, the metabolites of urinary microorganisms are also involved in the composition of urinary metabolites. Therefore, only analysing the correlation between urinary metabolites and diseases will result in large individual differences. Combined microbiome and metabolomics analysis of the characteristics of urinary microorganisms and metabolites in IC/BPS patients and the correlation analysis of differential urine microorganisms and differential metabolites will have more clinical significance.

Previous studies on the urinary microbiota of IC/BPS patients mostly focused on women. In our long-term outpatient work, we found that there were also many male IC/BPS patients, and there was a trend of gradual increase. Therefore, 11 male and 19 female IC/BPS patients were included in this study. The healthy control group consisted of 10 males and 20 females with matched demographic characteristics. Our results showed that compared with the healthy control group, there was no significant difference in α diversity in the urine of IC/BPS group patients (*P* > 0.01), but there was a significant difference in β diversity (*P* < 0.01). There were differences in the urinary microbiota between males and females in the IC/BPS group and the healthy control group, and the differences in bacterial genera of different sexes between the groups were significantly greater than the differences between males and females in the same group, suggesting that the urinary microflora structure of IC/BPS patients changed greatly. This study found that the abundances of *Lactobacillus, Acinetobacter, Brevibacterium, Pseudomonas, Stenotrophomonas,* and *Azospirillum* in the urine of IC/BPS patients were significantly lower than those of the control group, while the abundances of *Ralstonia, Bradyrhizobium, Sphingomonas,* and *Bosea* were significantly higher than those of the control group (*P* < 0.01). This study not only confirmed the findings of previous studies but also found many new differential microorganisms. The reason for the difference may be related to the fact that the patients included in this study all had severe lower urinary tract symptoms. This may also be because some male IC/BPS patients were included in this study, while most previous studies focused on female IC/BPS patients. In addition, different databases may be referenced in the analysis of different studies, which can also lead to differences in results. In this study, the SILVA (Version 132) database was used for taxonomic assignment to 16S rRNA reads and PICRUSt analysis.

Combining the findings of previous studies and the inflammatory mechanism of IC/BPS, we speculated that the differential genera *Lactobacillus* may be closely related to the inflammatory response of IC/BPS after analysing the differential genera identified by 16S rRNA sequencing one by one. *Lactobacillus* is a probiotic that may have beneficial effects on human health by regulating lipid metabolism, resisting oxidation, resisting the invasion by exogenous pathogens, and inhibiting the growth of pathogenic bacteria [18–20]. A large number of previous studies have shown that the pathogenesis and progression of IC/BPS are related to immunity, and the activation and degranulation of mast cells can promote the secretion of inflammatory factors such as interleukin-6 and TNF-α, which in turn promote the inflammatory response of IC/BPS [21–23]. Furuno T et al. found that a *Lactobacillus* strain (L. *kefiranofaciens*) could exert an immunomodulatory effect by reducing the level of mast cell degranulation [24]. The results of both previous studies and our study that found a low abundance of lactobacilli in the urine of IC/BPS patients sparked our interest: could decreased abundance of lactobacilli be a cause of increased levels of mast cell degranulation, interleukin-6, TNF-α and other inflammatory factors in IC/BPS patients? Could inhibition of mast cell degranulation by lactobacilli supplementation be a potential target for IC/BPS treatment? In addition, our study found a significantly higher abundance of other genera such as *Ralstonia* and *Sphingomonas* in the IC/BPS group than in the control group. *Ralstonia* is a low pathogenic microorganism, and its enrichment is associated with infectious diseases such as cystic fibrosis of the lung, osteomyelitis, and meningitis [25, 26]. *Sphingomonas* is an opportunistic pathogen, an emerging pathogen of hospital-acquired infections, and has previously been found to be the dominant genus in thymomas [27, 28]. However, their relevance to IC/BPS has not been reported in studies.

The results of nontargeted metabolomics analysis showed that metabolites such as purine, allantoin, and o-hydroxyphenylacetic acid were significantly higher in the IC/BPS group than in the control group, and the increase in these metabolites may potentially increase the burden on the kidney and cause damage to renal function. Allantoin, a metabolite of purine, is an excellent biomarker of oxidative stress in humans [29]. Another study found that allantoin was a sensitive marker of renal ischaemia–reperfusion injury in kidney transplant patients [30]. Again, after analysing all the differential metabolites one by one in the context of the inflammatory mechanisms of IC/BPS and the results of previous studies, we focused on theophylline, which is reduced in the urine of patients with IC/BPS. Theophylline is an anti-inflammatory substance, and previous studies have shown that theophylline can reduce the levels of IL-6, COMP, JAK2, STAT3, RANKL, iNOS, and eNOS by modulating the JAK/STAT/RANKL signaling pathway, thereby alleviating the inflammatory response in rheumatoid arthritis [31]. JAK/STAT signalling pathway inhibitors (microRNA-495, microRNA-132) can inhibit the JAK/STAT signalling pathway by downregulating JAK3, inhibiting the activation of mast cells, and the secretion of IL-6, NO, IFN-γ, TNF-α, and other inflammation factors relieving bladder fibrosis, and relieving the inflammatory response of ulcerative IC/BPS [32, 33]. Therefore, whether theophylline can also alleviate the clinical symptoms and bladder inflammation of IC/BPS by inhibiting the JAK/STAT signalling pathway is a new target for further research in this direction.

Most of the current studies on urinary microbial and metabolite alterations in IC/BPS are still preliminary explorations of the differences from the control group, and few involve in-depth studies of the mechanism. Although some studies have explored the combination of certain differential microorganisms and/or metabolites as diagnostic markers for IC/BPS, their sensitivity, and specificity still require further clinical validation [7, 13]. Considering that the excretion of urinary microorganisms may also influence the composition of urinary metabolites, our study integrated differential microorganisms with differential metabolites and performed correlation analyses. We identified some differential microbial-metabolite pairs, such as a negative correlation between the abundance of *Sphingomonas* and levels of theophylline, and a positive correlation between the abundance of *Lactobacillus* and *Gardnerella* and levels of theophylline. These differential microbial-metabolite associations point to potential targets for further exploration of the specific mechanisms by which urinary microbes and metabolites are involved in the inflammatory response to IC/BPS.

It should be noted that this study also has some limitations. First, the 16S rRNA sequencing method we employed can be used to identify microbes to only the genus level, and metagenomic sequencing was needed to identify the species level; the untargeted metabolomics analysis we used cannot obtain accurate differential metabolite concentrations, and targeted metabolomics analysis is necessary to accurately detect the concentration of target metabolites in future research. Second, the number of cases we included was small, and metabolites in urine were easily affected by local dietary habits, water quality, and urinary microbiota. Therefore, the results of this study need to be further confirmed by multicentre studies involving more patients from different regions.

## Conclusion

This study reveals the urinary microbial and metabolite profiles of IC/BPS patients, identifying some microorganisms and metabolites that differ in the urine of the IC/BPS group versus healthy controls, as well as between male and female patients. Combined with the results of the correlation analysis, we propose several urinary microorganisms and metabolites that may influence the inflammatory response to IC/BPS. Our preliminary findings provide a reference and preliminary basis for further studies on the mechanism of specific microorganisms and metabolites involved in the inflammatory response of IC/BPS.

## Supplementary Information

**Additional file 1: Table S1.** 16S rRNA sequencing information of IC/BPS patients and healthy controls.

**Additional file 2: Table S2**. Urinary differential metabolites in IC/BPS group and control group.

**Additional file 3: Table S3**. Qualitative and Quantitative results of urinary metabolites in IC/BPS group and control group by positive ion mode.

**Additional file 4: Table S4.** Qualitative and Quantitative results of urinary metabolites in IC/BPS group and control group by negative ion mode.

## Data Availability

The datasets used and/or analysed during the current study are available from the corresponding author upon reasonable request.

## Abbreviations

IC/BPS: Interstitial Cystitis/Bladder Pain Syndrome
16S rRNA: 16S Ribosomal RNA
LC–MS: Liquid Chromatograph-Mass Spectrometer
OTU: Operational Taxonomic Unit
BMI: Body Mass Index
VAS: Visual Analogue Scale for Pain
PUF: Patient Symptom Questionnaire for Pelvic Pain, Urgency and Frequency
ICSI: Interstitial Cystitis Symptom Index
ICPI: Interstitial Cystitis Problem Index Score
SDS: Self-Rating Depression Scale
SAS: Self-Rating Anxiety Scale
