# Supplementary material for: Integrated microbiome and metabolome analysis reveals novel urinary microenvironmental signatures in interstitial cystitis/bladder pain syndrome patients
Source: J Transl Med. 2023 Apr 19;21:266. doi: 10.1186/s12967-023-04115-5 (PMC10114403; doi:10.1186/s12967-023-04115-5)
Supplement: Supplementary file 1 — Additional file 1: Table S1. 16S rRNA sequencing information of IC/BPS patients and healthy controls. [file 12967_2023_4115_MOESM1_ESM.docx]

| **Additional Table 1. 16S rRNA sequencing information of IC/BPS patients and healthy controls** | | | | | | | | | |
| --- | --- | --- | --- | --- | --- | --- | --- | --- | --- |
| **Sample Name** | **Raw Tags** | **Effective Tags** | **Effective Ratio (%)** | **Total length** | **Max length** | **Min length** | **N50** | **N90** | **OTUs** |
| IC1-1 | 124455 | 120528 | 91.71 | 54675091 | 477 | 202 | 466 | 441 | 275 |
| IC1-2 | 125433 | 121016 | 90.8 | 54973124 | 478 | 205 | 466 | 441 | 312 |
| IC1-3 | 112933 | 108639 | 90.2 | 49285819 | 478 | 205 | 466 | 441 | 272 |
| IC1-4 | 123862 | 120152 | 89.43 | 54471043 | 474 | 204 | 460 | 441 | 800 |
| IC1-5 | 114951 | 109733 | 88.62 | 49667601 | 474 | 205 | 449 | 441 | 668 |
| IC1-6 | 116826 | 113672 | 90.54 | 51146959 | 474 | 202 | 443 | 441 | 562 |
| IC1-7 | 125091 | 119890 | 88.79 | 54343013 | 474 | 205 | 460 | 441 | 675 |
| IC1-8 | 117234 | 114077 | 89.44 | 51693790 | 474 | 203 | 460 | 441 | 645 |
| IC1-9 | 117501 | 113507 | 88.85 | 51329452 | 474 | 206 | 449 | 441 | 578 |
| IC1-10 | 123693 | 119998 | 91.2 | 53455799 | 474 | 205 | 441 | 441 | 1127 |
| IC1-11 | 114927 | 110069 | 88.04 | 49226560 | 474 | 204 | 444 | 441 | 651 |
| IC2-1 | 123385 | 119711 | 92.12 | 54397616 | 478 | 205 | 466 | 441 | 267 |
| IC2-2 | 115012 | 111094 | 91.22 | 50313054 | 478 | 205 | 443 | 441 | 284 |
| IC2-3 | 119492 | 106198 | 81.33 | 48385138 | 478 | 206 | 460 | 441 | 318 |
| IC2-4 | 125264 | 120590 | 91.39 | 54635650 | 478 | 202 | 464 | 441 | 277 |
| IC2-5 | 127536 | 122310 | 89.69 | 55559795 | 478 | 205 | 458 | 441 | 347 |
| IC2-6 | 122874 | 119236 | 92.21 | 54196118 | 478 | 205 | 466 | 441 | 282 |
| IC2-7 | 119941 | 112854 | 88.26 | 51510891 | 478 | 202 | 466 | 441 | 523 |
| IC2-8 | 116609 | 112716 | 91.37 | 51090655 | 478 | 205 | 465 | 441 | 272 |
| IC2-9 | 113787 | 100012 | 80.67 | 45361963 | 474 | 203 | 460 | 441 | 960 |
| IC2-10 | 109993 | 104202 | 86.21 | 47301964 | 474 | 202 | 460 | 441 | 1217 |
| IC2-11 | 125692 | 118498 | 87.09 | 53710962 | 474 | 202 | 460 | 441 | 638 |
| IC3-1 | 117896 | 114015 | 91.13 | 51742991 | 478 | 202 | 466 | 441 | 279 |
| IC3-2 | 120253 | 116124 | 91.55 | 52592664 | 478 | 202 | 446 | 441 | 295 |
| IC3-3 | 128991 | 124168 | 90.59 | 56522732 | 478 | 202 | 466 | 441 | 303 |
| IC3-4 | 117152 | 113534 | 92.04 | 51431770 | 477 | 206 | 446 | 441 | 263 |
| IC3-5 | 129601 | 125588 | 91.76 | 57016315 | 477 | 205 | 466 | 441 | 284 |
| IC3-6 | 113235 | 111898 | 92.45 | 52102555 | 472 | 230 | 466 | 466 | 282 |
| IC3-7 | 119767 | 112606 | 87.76 | 50660648 | 474 | 207 | 455 | 441 | 490 |
| IC3-8 | 111617 | 108394 | 90.18 | 49509350 | 474 | 207 | 466 | 441 | 335 |
| N1 | 121842 | 116171 | 88.56 | 52903939 | 474 | 204 | 462 | 441 | 1881 |
| N2 | 120928 | 114741 | 88.17 | 51774594 | 474 | 202 | 448 | 441 | 529 |
| N3 | 125600 | 123198 | 90.39 | 56777081 | 474 | 205 | 466 | 443 | 478 |
| N4 | 122441 | 118342 | 88.58 | 53726158 | 474 | 204 | 460 | 441 | 1421 |
| N5 | 122756 | 116179 | 87 | 52949367 | 474 | 201 | 461 | 441 | 689 |
| N6 | 118577 | 114763 | 88.79 | 52059158 | 474 | 205 | 460 | 441 | 611 |
| N7 | 119114 | 116254 | 89.91 | 53100789 | 474 | 205 | 461 | 441 | 628 |
| N8 | 122087 | 117397 | 89.47 | 53284309 | 474 | 201 | 461 | 441 | 1202 |
| N9 | 113490 | 108379 | 87.61 | 49050328 | 474 | 202 | 460 | 441 | 1040 |
| N10 | 116105 | 110688 | 88.09 | 50313238 | 474 | 201 | 451 | 442 | 1071 |
| N11 | 118795 | 116558 | 90.86 | 53588206 | 474 | 203 | 466 | 442 | 598 |
| N12 | 117199 | 113483 | 89.79 | 51447721 | 474 | 205 | 460 | 441 | 1096 |
| N13 | 122403 | 117139 | 87.88 | 53494302 | 474 | 202 | 461 | 441 | 770 |
| N14 | 123475 | 121162 | 90.64 | 56153467 | 478 | 211 | 466 | 443 | 140 |
| N15 | 118777 | 115690 | 91.6 | 53052222 | 478 | 205 | 466 | 441 | 215 |
| N16 | 117855 | 115147 | 91.32 | 53433440 | 478 | 206 | 467 | 465 | 192 |
| N17 | 127724 | 125225 | 91.4 | 58077617 | 478 | 202 | 466 | 448 | 145 |
| N18 | 115664 | 112821 | 91.73 | 52301787 | 478 | 205 | 466 | 449 | 200 |
| N19 | 125196 | 121885 | 91.17 | 56324378 | 478 | 201 | 466 | 442 | 208 |
| N20 | 113417 | 109999 | 89.31 | 49740920 | 478 | 201 | 448 | 444 | 177 |
| N21 | 115027 | 112867 | 91.36 | 52214105 | 478 | 203 | 466 | 444 | 162 |
| N22 | 117678 | 114344 | 90.12 | 52137570 | 478 | 205 | 461 | 441 | 192 |
| N23 | 122252 | 118243 | 90.54 | 53721746 | 478 | 202 | 461 | 441 | 204 |
| N24 | 115381 | 113114 | 92 | 52619325 | 478 | 230 | 466 | 466 | 164 |
| N25 | 112850 | 110244 | 89.88 | 50829686 | 478 | 205 | 466 | 443 | 137 |
| N26 | 121033 | 118489 | 91.5 | 54917426 | 478 | 222 | 466 | 461 | 174 |
| N27 | 113330 | 111199 | 92.26 | 51735500 | 478 | 222 | 466 | 466 | 161 |
| N28 | 117216 | 115327 | 91.83 | 53088511 | 478 | 205 | 466 | 448 | 118 |
| N29 | 124432 | 121766 | 91.73 | 56481210 | 478 | 217 | 466 | 461 | 207 |
| N30 | 119158 | 115674 | 90.53 | 53212218 | 478 | 206 | 461 | 450 | 193 |
