# Supplementary material for: Integrated microbiome and metabolome analysis reveals novel urinary microenvironmental signatures in interstitial cystitis/bladder pain syndrome patients
Source: J Transl Med. 2023 Apr 19;21:266. doi: 10.1186/s12967-023-04115-5 (PMC10114403; doi:10.1186/s12967-023-04115-5)
Supplement: Supplementary file 2 — Additional file 2: Table S2. Urinary differential metabolites in IC/BPS group and control group. [file 12967_2023_4115_MOESM2_ESM.docx]

| **Additional Table 2. Urinary differential metabolites in IC/BPS group and control group** | | | | |
| --- | --- | --- | --- | --- |
| **Differential metabolite names** | **log2_FC (IC/control)** | **Pvalue** | **fdr** | **vip** |
| 2-Phosphinomethylmalate | -1.806640836 | 0.016765372 | 0.280009898 | 1.259040505 |
| CE(14:0) | -1.224665993 | 0.001399693 | 0.101907599 | 1.350629596 |
| Trimethylselenonium | -0.945842304 | 0.042800925 | 0.419243794 | 11.30386682 |
| PE-NMe(14:0/18:2(9Z,12Z)) | -0.911593909 | 0.002693173 | 0.106930889 | 1.859838921 |
| Fosetyl | -0.815467485 | 0.02178928 | 0.317455956 | 6.055571644 |
| Pyrogallol-2-O-glucuronide | -0.800445414 | 0.043498238 | 0.420066318 | 1.115936979 |
| D-4&apos;-Phosphopantothete | -0.627295397 | 0.044261318 | 0.425804014 | 2.256450411 |
| 4-Bromo-3,5-cyclohexadiene-1,2-dione | -0.591744101 | 0.005261719 | 0.142603907 | 2.089465819 |
| 1-Methylhypoxanthine | -0.92074822 | 0.018048373 | 0.286106436 | 1.855572537 |
| 2,6 Dimethylheptanoyl carnitine | -0.839786582 | 0.032372433 | 0.374287689 | 3.186180095 |
| all-trans-Retinoic acid | -0.737924256 | 0.025664009 | 0.334295273 | 1.231568182 |
| Dihydro-2-methoxy-2-methyl-3(2H)-thiophenone | -0.865694218 | 0.005222884 | 0.142603907 | 1.32306962 |
| PE-NMe2 | -1.313872272 | 0.002182786 | 0.103279417 | 2.173359977 |
| Theophylline | -1.878678735 | 0.004494747 | 0.130218509 | 1.800207648 |
| N-Methyl-N&apos;-nitro-N-nitrosoguanidine | 0.705570801 | 0.025330132 | 0.33339215 | 1.805055432 |
| 3-hydroxy-2-isobutyrate | 0.866253561 | 0.035326735 | 0.389695667 | 2.038766158 |
| N-Lactoyl ethanolamine phosphate | 3.081777176 | 0.001179435 | 0.101907599 | 1.379272989 |
| 4-Bromophenylthiourea | 4.144682708 | 0.004326203 | 0.126792973 | 1.417111274 |
| Potassium dichromate | 4.459895209 | 0.007295998 | 0.174308665 | 1.177609534 |
| D-Mannitol 1-phosphate | 6.702416295 | 0.000732879 | 0.080924584 | 3.090418476 |
| D-Ribose 5-phosphate | 6.783813432 | 0.001907482 | 0.101907599 | 3.492586798 |
| 4-[2,2-Dichloro-1-(4-methoxyphenyl)ethenyl]phenol | 6.966473873 | 0.007559167 | 0.175476554 | 2.878318739 |
| MC-7181 | 8.74630121 | 0.000171575 | 0.033265722 | 1.219988317 |
| Potassium dichromate | 12.25387013 | 0.000376179 | 0.057464213 | 1.483668186 |
| Perfluorohexane sulfonic acid | 13.03171749 | 0.045931874 | 0.434413842 | 1.174200894 |
| Cephalosporin C | 9.152252772 | 0.025016118 | 0.332735664 | 1.168923333 |
| Etrimfos | 1.066375815 | 2.24E-05 | 0.011304411 | 1.107488736 |
| Nortriptyline | 9.826542773 | 0.004288099 | 0.126411156 | 2.783238362 |
| Sorbitol-6-phosphate | 2.041595147 | 0.042566247 | 0.419094632 | 1.261331391 |
| 3,4-Dihydro-6-hydroxy-2,5,7,8-tetramethyl-2H-1-benzopyran-2-carboxylic acid | -1.922799672 | 0.000102617 | 0.105119002 | 1.81119724 |
| 1,3,7-Trimethyluric acid | -1.615854374 | 0.031561522 | 0.510709104 | 1.110389844 |
| Erythrono-1,4-lactone | -1.186800094 | 0.016425822 | 0.404506998 | 1.623518574 |
| 1D-myo-Inositol 1,5-bis(diphosphate) 2,3,4,6-tetrakisphosphate | -0.869328436 | 0.002423196 | 0.173549014 | 1.226375167 |
| 2-Bromo-1H-indole-3-carboxaldehyde | -1.643210532 | 0.049629594 | 0.566263437 | 1.54652758 |
| Disodium malate | -1.679939088 | 0.030140763 | 0.510709104 | 3.191277844 |
| Disodium malate | -0.963962146 | 0.020229322 | 0.446433007 | 1.703840273 |
| PE-NMe(11M3/9D3) | -1.253420096 | 0.001827416 | 0.173549014 | 1.516857512 |
| PE-NMe(15:0/20:5(5Z,8Z,11Z,14Z,17Z)) | -0.726646261 | 0.004861966 | 0.228977912 | 1.31879023 |
| Soyacerebroside I | -1.190887459 | 0.001850853 | 0.173549014 | 2.512977885 |
| Plutonium | 1.584919179 | 0.027387184 | 0.496897803 | 4.219395472 |
| 1-Deoxy-D-xylulose 5-phosphate | 3.09109202 | 0.030167466 | 0.510709104 | 1.789513479 |
| 1-Methylguanine | 4.19648259 | 0.000485977 | 0.173115558 | 1.080796134 |
| Allantoin | 0.888567819 | 0.009988618 | 0.317498573 | 2.915839547 |
| Ortho-Hydroxyphenylacetic acid | 0.765816125 | 0.028096027 | 0.500625199 | 3.006322927 |
